# Cellular agriculture in the UK: a review

**DOI:** 10.12688/wellcomeopenres.15685.2

**Published:** 2020-10-12

**Authors:** Neil Stephens, Marianne Ellis

**Affiliations:** 1 Social and Political Sciences, Brunel University London, Uxbridge, UB8 3PH, UK; 2 Department of Chemical Engineering, University of Bath, Bath, BA2 7AY, UK

**Keywords:** Cellular agriculture, Cultured meat, Clean meat, Cell-based meat, Cultivated meat, UK, Palm oil

## Abstract

This review details the core activity in cellular agriculture conducted in the UK at the end of 2019, based upon a literature review by, and community contacts of the authors. Cellular agriculture is an emergent field in which agricultural products—most typically animal-derived agricultural products—are produced through processes operating at the cellular level, as opposed to (typically farm-based) processes operating at the whole organism level. Figurehead example technologies include meat, leather and milk products manufactured from a cellular level. Cellular agriculture can be divided into two forms: ‘tissue-engineering based cellular agriculture’ and ‘fermentation-based cellular agriculture’. Products under development in this category are typically valued for their environmental, ethical, and sometimes health and safety advantages over the animal-derived versions.

There are university laboratories actively pursuing research on meat products through cellular agriculture at the universities of Bath, Newcastle, Aberystwyth, and Aston University in Birmingham. A cellular agriculture approach to producing leather is being pursued at the University of Manchester, and work seeking to produce a palm oil substitute is being conducted at the University of Bath. The UK cellular agriculture companies working in the meat space are Higher Steaks, Cellular Agriculture Ltd, CellulaRevolution, Multus Media and Biomimetic Solutions. UK private investors include CPT Capital, Agronomics Ltd, Atomico, Backed VCs, and Breakoff Capital. The UK also has a strong portfolio of social science research into diverse aspects of cellular agriculture, with at least ten separate projects being pursued over the previous decade. Three analyses of the environmental impact of potential cellular agriculture systems have been conducted in the UK. The first dedicated third-sector group in this sector in the UK is Cultivate (who produced this report) followed by Cellular Agriculture UK. International groups New Harvest and the Good Food Institute also have a UK presence.

## Introduction: What is cellular agriculture?

Cellular agriculture is an emergent field in which agricultural products—most typically animal-derived agricultural products—are produced through processes operating at the cellular level, as opposed to (typically farm-based) processes operating at the whole-organism level. This review details the cellular agriculture landscape in the UK at the time of publishing, providing an overview of key actors in the sector from a range of backgrounds, including university and corporate laboratory research, private investors, social science, Life Cycle Analyses, and policy work. The aim of the review is to collate information on the UK context in a form that is not currently available in the public domain. It provides a brief overview of the key groups and individuals involved, and identifies each by the sector in which they operate. The goal is to provide a brief and accessible review that, where possible, signposts readers to further sources that can be pursued for additional information. As such it is not intended as a detailed overview of the technical aspects of the work discussed. Instead, the review provides a basis for subsequent discussion both within and beyond the UK about activity in the country. We anticipate it will have value for policy-makers, academics, NGOs, and other actors interested in learning about and engaging with cellular agriculture in the UK, and will form a building block upon which subsequent discussion can be conducted.

The term cellular agriculture was first coined in 2015 by Isha Datar, Executive Director of US-based 3 ^rd^ sector group New Harvest. Potential future products bracketed under the label cellular agriculture include meat produced though tissue engineering (variously known as cultured meat, clean meat, cell-based meat and cultivated meat (referred to herein as CM)), and animal-derived products such as milk, leather and egg white produced through recombinant DNA fermentation techniques ( Datar *et al.*, 2016; Stephens *et al.*, 2018). As these examples suggest, cellular agriculture is typically divided into two types, based on the technology form used. The first has been called ‘tissue engineering-based cellular agriculture’ ( Stephens *et al.*, 2018) and involves taking cells from live (or recently deceased) animals and culturing these cells so as to control their cell proliferation and differentiation to direct the formation of increasing quantities of a desired cell type (e.g. muscle and fat for meat, skin for leather). We use this term to capture work on both structured and unstructured products (e.g. mincemeats and full tissue meats). The second type of cellular agriculture has been termed ‘fermentation-based cellular agriculture’ (ibid) and involves genetically modifying typically bacteria, yeast or algae by adding recombinant DNA so that when they are fermented in sugars they produce organic molecules that can subsequently be processed to biofabricate familiar products such as milk and leather.

While the term cellular agriculture is less than five years old, the technologies it describes have a longer history. In terms of tissue engineering-based cellular agriculture, the first work to increase the mass of *in vitro* muscle under laboratory conditions happened around the millennium ( Benjaminson *et al.*, 2002; Catts & Zurr, 2002), with further work in the following decade ( Puy *et al.*, 2010; Wilschut *et al.*, 2008). In terms of fermentation-based cellular agriculture, some suggest the historical lineage can be drawn as far back as industrially-produced rennet used in manufacturing cheese, which has used recombinant DNA techniques since the 1990s to replace enzymes taken from ruminant mammals, typically after their slaughter. However, manufacturers using this technique have not adopted the term cellular agriculture and do not feature within the emergent cellular agriculture community, so we do not include rennet in our review. Similarly, some products that are naturally produced in plants can be made using fermentation, such as flavour molecules and oils like vanilin, but these have not generally be classed as cellular agriculture to date. It could be argued that they fall under the same umbrella if their goal is the same (i.e. sustainable production), as is the case of the palm oil substitute discussed later.

The call for cellular agriculture is generally motivated by a set of related concerns about the impacts of animal agriculture as it exists today and the challenges of the increased global population in the coming decades. Cellular agriculture can be said to be directed at addressing UN Sustainable Development Goal Two (Zero Hunger) and Goal Twelve (Responsible Consumption and Production). While the exact form the potential contribution of cellular agriculture may take varies from case to case, the recurrent themes are a concern with the environmental impact of animal agriculture (in terms of land use, greenhouse gas emissions, impact on biodiversity etc., see Bhat *et al.*, 2015; Mattick, 2018), as well as animal ethics concerns about livestock living conditions and slaughter ( Milburn, 2018; Schaefer & Savulescu, 2014), and the impact on human health of animal agriculture through issues such as animal-borne disease and antibiotic use ( Arshad *et al.*, 2017; Specht *et al.*, 2018). The view is that cellular agriculture will allow the continued production of familiar animal products while using either fewer or no animals in the process. The aim is that this would result in either reducing (or perhaps entirely replacing) animal agriculture, or that it would slow the increase in global use of animals in agriculture to meet global rising demand for animal products driven by population and wealth increases. This given, while there is optimism within the community, a number of substantial technical hurdles remain. This includes producing culture media at a lower cost; developing cell lines that have the capacity for indefinite propagation and have a high culture-efficiency to maximise product yield; developing reliable automation and bioreactor systems to enable scale-up that is net-zero carbon; and reproducing the taste, texture and nutritional profile of familiar peat products ( Stephens *et al.*, 2018; Thorrez & Vandenburgh, 2019).

In this review we suggest the common-use definition of cellular agriculture could be expanded to also include the cellular production of agricultural goods that are not sourced from animals, here reflecting upon the example of fermentation-based palm oil production (an active area of research in the UK). Intuitively this makes sense, as this fermentation-based work is producing agricultural products at the cellular level. This work also ties to environmental and animal welfare concerns, specifically around deforestation and its impact upon greenhouse gases and biodiversity, that are also seen expressed in different ways elsewhere within the cellular agriculture community. However, at this stage, we raise this as just a possibility, and do not seek to assert a new definition upon the field.

## Global context

The leading nations in cellular agriculture today arguably are the US, the Netherlands, and Israel, although work is conducted in numerous other countries, including the UK. In terms of CM, the first larger scale project was conducted in the Netherlands from 2005 onwards. One member of this initial consortia, Prof Mark Post of Maastricht University, went on to secure funding from Google co-founder Sergey Brin to produce the world’s first laboratory-grown hamburger, which was cooked and tasted at a press conference in London in 2013 ( O’Riordan *et al.*, 2017; Post, 2014). The interest this generated fed into a change of culture within the international CM community, as the first 10–15 years of largely university-based research shifted towards the emergence of a start-up culture ( Stephens *et al.*, 2019). While University research has continued, the focus has shifted to the swiftly increasing number of early stage companies in the area, seeking and securing venture capital seed funding for their work. Among the highest profile in the US are Memphis Meats, who were the first CM company to secure series A funding of $17m, and the vegan-mayonnaise and liquid egg company Just (formally Hampton Creek) who have also established a CM R&D initiative. Others include Mission Barns, Wild Type, and Bluefin tuna-focused Finless Foods. Outside of the US, the Netherlands has remained a key site with Post following his burger press conference with the establishment of a start-up, Mosa Meat, which in 2018 announced a funding round of €7.5m. A second company, Meatable, also runs out of Leiden. Israel also has a strong base, with companies including Future Meat Technologies and Aleph Farms, who recently completed a funding round of $11.65m. Other examples include Japan-based Integriculture, Singapore-based Shiok Meats, and Canada-based Appleton Meats. As private entities, it is not always clear exactly what proprietary technology each company is developing, or how advanced their technology is.

In terms of fermentation-based cellular agriculture, the international context is dominated by US companies, particularly those based in the San Francisco Bay area. Key examples include Geltor, who produce gelatin, Clara Foods, who produce egg white, and both Perfect Day and New Culture, who produce animal-free dairy products. Outside of California, the most prominent company is Modern Meadow, who ferment collagen to use in manufacturing leather-like products. Modern Meadow were initially the first company to work on CM, but later focused exclusively on leather. Beyond the US, the main example would be Japan-based Spiber, who make spider silk. Further information on the global context can be found in State of the Industry Reports for CM and plant-based meats, eggs and dairy from the Good Food Institute.

Histories of cellular agriculture often include two key UK-related components, both related to CM. The earliest is a regularly repeated quotation from Winston Churchill in a 1931 article titled ‘Fifty Years Hence’, in which he states “[w]e shall escape the absurdity of growing a whole chicken in order to eat the breast or wing, by growing these parts separately under a suitable medium” ( Churchill, 1931). Typically, people from the community follow the quotation by noting it is taking longer than Churchill predicted, but the trajectory was correct. The second UK-related historical milestone is the 2013 London press conference in which Post and his team at Maastricht University unveiled the world’s first cultured burger, which had been grown in his laboratory in the Netherlands and transported to the UK just before. The burger was a proof of concept as opposed to a product launch, as it was reported to have cost around $300,000. It was tasted by two independent food journalists from Austria and the US, after being cooked by chef Richard McGeown, patron of Couch's Great House Restaurant in Cornwall, UK ( O’Riordan *et al.*, 2017; Post, 2014).

## Review methodology

This review is rooted in a ten-year social science project conducted by N.S. This project has involved over 50 interviews with experts internationally in CM, as well as attendance at key meetings and media analysis of reporting on the subject. N.S. and M.E. are part of a group that co-founded an organisation named ‘Cultivate’ in 2016 to act as a multi-voiced forum for discussing issues around cellular agriculture in the UK. Cultivate organises an annual event to bring together the UK cellular agriculture community. During its November 2018 event a draft document produced by N.S. on UK activity in this area was circulated and commented upon by those present. After this, N.S. followed up the suggestions made in that discussion and conducted further desk-based research and direct contact with groups involved to develop the work reported here. This exercise was addressed again during Cultivate event in November 2019 to up-date and finalise the review.

As noted above, cellular agriculture is an emerging field, and much of the leading research has been conducted within private companies that do not typically publish their research. As such, this review includes fewer references to peer-reviewed sources than would be the case in a typical review article. This is by necessity, as there are only a limited number of peer-reviewed papers available on this novel area of research. Subsequently this review combines references to peer-reviewed research with grey literature found in reports, institutional websites, and media reporting.

We note that we ourselves are among the most active in this field in the UK, and as such we review our own work as part of this text. We also note that cellular agriculture can sometimes seem a fast moving field, with new entrants appearing frequently. Often in the commercial sphere such entities operate in what is termed ‘stealth mode’, to indicate a low profile. As such, it is possible our account may miss some UK activity, and can only claim to capture the context as we know it to be at the time of publication.

In the following sections we review key aspects of UK cellular agriculture activity, focusing upon different clusters of activity in turn.

### University laboratory research

The most active university in the UK is the University of Bath. Dr Marianne Ellis and Dr Chris Chuck are the bioprocessing strand lead and director, respectively, in the Centre for Integrated Bioprocessing Research (CIBR). The Ellis group focuses on tissue engineering-based cellular agriculture; the Chuck group focuses on fermentation-based cellular agriculture, specifically production of a palm oil substitute from yeast. Ellis began her research career in regenerative medicine and has applied her bioprocess design techniques to the expansion of muscle cells for CM. Given her experience with *in vitro* liver models ( Luetchford *et al.*, 2018; Storm *et al.*, 2016) and her early-career work on scaffold development for mesenchymal stem cell expansion ( Morgan *et al.*, 2007), she has positioned herself to develop platform technologies for a wide range of tissue engineered cellular agriculture products, specifically based around scalable bioreactor design ( Allan *et al*., 2019). She has a research group, with funding from New Harvest amongst others, developing bioreactors and scaffolds for CM, and gives Chemical Engineering undergraduate students the opportunity to carry out major project work in this space, having now supervised over 30 Masters of Engineering student projects on the topic of cultured meat bioprocess design. The work combines tissue engineering with established biotechnology process design with the addition of novel approaches to scaling up tissue engineering cultures. Focus is on bioreactor configuration; given the early stage of this work, much effort is going into understanding the metabolic stoichiometry, i.e. how much raw material is consumed by the cells to produce a given amount of protein as well as the waste products. This is intimately linked to efficient scale up due to it being the basis for media recipes and amounts required, which is likely to be affected by cell type and culture conditions.

Aberystwyth University has recently started working on cellular agriculture via a PhD student co-funded by M.E.’s start-up, Cellular Agriculture Ltd, the Institute of Biology, Environment and Rural Sciences (IBERS) at Aberystwyth University and the Pedigree Welsh Pig Society. The project is examining cell sourcing and harvest for cultured pig meat. To our knowledge this is the first and only study in the world exploring the properties of primary porcine cells to find the most efficient for CM production. This type of research is commonplace in traditional meat production, albeit for the whole animal, and it follows that there will be particular breeds whose cells are more conducive to the bioreactor culture environment than others, thus leading to a more efficient production process, like the broiler chicken, and even a customer-preferred source likened to prime cuts of meat like Aberdeen Angus beef.

Also, in 2019, US-led third sector group the Good Food Institute provided $210,088 funding to Petra Hanga, lecturer in Biological Engineering at Aston University, to work on bioprocessing and scale-up. Her focus is upon microtissues composed of fat and muscle in a scalable bioreactor platform. Working with bovine mesenchymal stem cells, the project aims to optimise protocols for increased cell production.

From 2018 until late 2019, US-based third sector group New Harvest have also been funding Dr Ricardo M. Gouveia at Newcastle University to investigate how substrate curvature effects the migration, proliferation and self-organisation of cells within a matrix, and how controlling this could support targeted bio-fabrication of tissues that reproduce the texture of meat. This work is based within the lab of Prof Che J. Connon, which is also connected to the start-up CellulaREvolution discussed in the next section.

At the University of Cambridge, academic neurosurgeon Dr Mark Kotter has developed work on human induced pluripotency stem cells to produce neurons and skeletal myocytes for biomedical purposes ( Pawlowski *et al.,* 2017). Kotter’s new technology – named OPTi-OX – has been licensed to the Netherlands-based company Meatable, who are using it to develop a CM system. Kotter co-founded Meatable, along with Krijn de Nood and Daan Luining, in 2018, with its aims including producing a pork prototype.

Returning to the University of Bath, and moving away from CM, Chuck has been working on the scale up of oleaginous yeasts grown on waste resources for the production of a palm oil substitute ( Parsons *et al.*, 2018; Whiffin *et al.*, 2016). Funded by a £3.9m grant from the EPSRC and Innovate UK, this MP² Project is a collaboration between the University of Bath, University of York, Croda, and AB Agri. They seek to create a sustainable biorefinery that uses food waste biomass that has been broken down with a one-step and additive-free microwave technology to hydrolyse the materials into fermentable sugars. Then, using oleaginous yeast as a platform mechanism, the MP² Project group seek to develop a pilot-industrial scale bioreactor to produce larger quantities of single cells oils that can operate as a palm oil substitute for some purposes. Their current research involves optimising the yeast strain being used, and assessing the mechanics and economics of a scale-up system. The long-term goal is to produce a palm oil substitute—the world’s most widely used oil crop—with a system involving less deforestation and the associated habitat destruction.

Finally, moving away from food but remaining with cellular agriculture, in 2019 an Engineering and Physical Sciences Research Council-funded PhD project commenced at the University of Manchester. Supervised by Dr Celina Jones, Dr Olga Tsigkou and Dr Lucy Bosworth, the project aims to pair a synthetic scaffold with 3D cell culture techniques to produce a new uniformed textile-cell construct, or ‘leather’. The vision is to enable traditional fibre-scaffolds to be transformed into unique fabrics using textile processes. These biodegradable fabrics would then be cultured with fibroblasts, which should secrete extracellular matrix proteins (including collagen and elastin), and eventually be modified to be comparable to the dermis layer of the skin. This ‘artificial’ skin would then be subjected to traditional tanning processes, minus a number of previously essential steps, in an attempt to create a mechanically stable, uniform leather material.

### Companies

The most visible CM company in the UK is Higher Steaks, founded by Benjamina Bollag (CEO), Dr Stephanie Wallis (CSO), and Prof David Hay (Scientific Director) in 2017. Higher Steaks is a ‘full stack’ company, meaning its focus is upon producing a consumer ready CM product, as well as working on all the intermediary steps in a vertically integrated form. They are developing a technology that could use skin biopsies or blood samples from pigs to which an induced pluripotency technique is applied to create cells that could produce any type of tissue including muscle and fat for use in pork products. Pork has been chosen as the initial focus as porcine biology is close to human biology, allowing biomedical insights to be more easily translated, and because of Higher Steaks’ concern that pigs in the meat industry have a higher exposure to antibiotics than cattle. However, they also expect their technology to be applicable to other species in the future. Like many full stack CM start-ups, they are also working to develop methods for reducing the cost of the media in which their cells are grown, as media is the highest costing input to the process. On this, they have already established culturing protocols that work sufficiently well without fetal bovine serum (an animal-derived blood product), but are continuing research efforts in this area. In mid-2019, the team was made up of Bollag and Wallis and a stem cell scientist, while Hay contributed in parallel to his role as the chair of the Tissue Engineering department at MRC Centre for Regenerative Medicine at the University of Edinburgh. They also have a team of advisors. Higher Steaks raised a pre-seed round and are currently preparing for their seed round and anticipate expanding once further capital is secured.

While Higher Steaks have been more visible, the first CM company to be established in the UK was Cellular Agriculture Ltd, founded in 2016 by Illtud Dunsford and Ellis. The company is in some regards distinct internationally: co-founder Dunsford’s background is in farming as the owner of a successful meat production and processing business set on the family farm of 300 years (Charcutier Ltd), giving the company an unusual grounding in traditional meat production. Cellular Agriculture Ltd has not adopted a ‘full stack’ business model and does not seek to produce meat themselves. Instead they are commercialising the bioprocess with focus on the bioreactor technology, developed in the Ellis laboratory at the University of Bath, to enable the industry to manufacture their products on a commercial scale. The company is leveraging its university contacts, and co-funds two PhD students alongside US-based 3 ^rd^ sector group New Harvest and the University of Bath for bioreactor design, and Aberystwyth University for cell sourcing and harvesting. It has also developed its own proof of concept bioreactor via InnovateUK funding. In mid-2019 they were preparing their seed round.

There have been, and continue to be, other companies with UK links active in the field. CellulaREvolution Ltd are a new spin out company from Newcastle University co-founded by Leo Groenewegen CEO, Dr Martina Miotto CSO and Prof. Che Connon CTO. The team work with peptides for multiple purposes. This includes developing methods for continuous bioprocessing in which cells automatically self-detach from their growing surface to allow other cells to subsequently grow in the same space. This substantially increases production yields, all within a serum-free environment whilst reducing media volume and footprint ( Miotto *et al*., 2017). Their research was originally developed for medical uses, particularly the cornea, but they are now exploring applications in both biomedicine and CM ( CellulaREvolution, 2018). In November 2019, CellulaREvolution announced a £380,000 investment via the North East Angel R&D programme, managed by Northstar Ventures.

Also recently established, Multus Media are seeking to produce animal-free, sustainable and cheap media for the CM industry. Their approach is to use genetically engineered yeast to produce mammalian cell growth factors. Based out of Imperial College Advanced Hackspace (ICAH) in London, the project is led by Kevin Pan with a team of 13 other scientists.

Another start-up, Biomimetic Solutions, is also exploring developing enabling technologies with applications in both CM and biomedicine. Starting in Brazil, the company moved to London in 2018 and participated in the RebelBio accelerator programme. Currently Biomimetic Solutions retains links to both Brazil and the UK. They have patented a scaffold called Nano3D that is edible and pH neutral that could provide a framework for muscle cells to grow into as CM is produced. The scaffold has been trialled by US CM company Finless Foods ( Benz, 2018).

### Private investors

The most active private funder of cellular agriculture work in the UK is CPT Capital, a dedicated investor in the alternative protein sector. It is run through the family office of Jeremy Coller, a successful financial executive. They have invested in both plant-based proteins (including famous names like Beyond Meat and Impossible Foods) as well as a string of cellular agriculture companies, including Geltor (gelatine), Perfect Day and New Culture (both dairy products), Modern Meadow and Vitro Labs Inc (both leather), and Blue Nalu, Aleph Farms, Memphis Meats, and Mosa Meat (all CM). While these are largely US-based companies, CPT Capital are now “ looking to expand the geographic representation” of their portfolio. They look for pre-seed to Series B stage companies with a view to long-term support.

The second most active investor in the sector that we are currently aware of is Agronomics Limited, who focus specifically on nascent modern foods that target environmental benefits. Their listed investments include BlueNalu, New Ages Meats, Shiok Meats and Meatable. Other single company investments from the UK include Atomico’s investment in Memphis Meats, Backed VC’s investment in the Dutch company Meatable, and Breakoff Capital’s investment in Finless Foods. Other UK-related investors include Richard Branson, who famously invested in Memphis Meats Series A round, and, as reported above, biotech incubator RebelBio (backed by global venture capital firm SOSV), who supported Biomimetic Solutions.

### Social science

The UK has a broad range of social science analyses of cellular agriculture. The earliest was an economic forecast produced by eXmoor pharma concepts (2008). This work was funded by the Dutch-led In Vitro Meat Consortium project, and predicted CM could be produced for €3300–3500 per tonne, compared to about €1800 per tonne for chicken meat.

At this time, sociologist Dr Neil Stephens began an extended project, still continuing today, tracking the long-term development of CM and the community that supports it. His early work identified the ontological ambiguity over what CM actually is—as meat, or as meat alternative, or even not as food at all—and has subsequently documented the technical and cultural moves that have sought to define its status and politics ( O’Riordan *et al.,* 2017; Stephens, 2010; Stephens, 2013; Stephens & Ruivenkamp 2016; Stephens *et al.,* 2018; Stephens *et al.,* 2019). Stephens’ work has received funding from the Economic and Social Research Council, the Wellcome Trust, and via a larger FP7 project (titled EPINET). It has stressed the symbolic work conducted by those within the cultured meat community to assert frames of reference that position the politics of cultured meat in specific ways.

Continuing the interdisciplinary theme, lawyer Dr Ludivine Petetin (2014), then of the University of Hull, published work identifying key questions about the politics and policy landscape of CM, before arguing EU regulation needed strengthening in response to CM, in order to strike a balance between ensuring risk management is accomplished effectively, while avoiding stifling innovation.

In more work funded by the Wellcome Trust, bioethicists Dr Owen Schaefer and Prof Julian Savulescu (2014), then of the University of Oxford, argued CM is permissible and worth promoting. They found potential moral objections – disrespect towards animals, reduction of happy animal numbers, and risks of cannibalism – are insufficiently serious to undermine the potential positives offered by the technology.

2014 also saw the first UK academic meeting dedicated largely to CM. Titled ‘The Ethics of In-Vitro Flesh and Enhanced Animals’ and hosted by Dr Jan Deckers in the small Northumberland Town of Rothbury, the two-day event featured a range of social science papers addressing the issue. Along with Stephens and Schaefer, another attendee was geographer Dr Alexandra Sexton, now of the University of Oxford and a co-founder of Cultivate. Funded initially by the ESRC, and then as part of the broader Wellcome Trust funded Livestock, Environment and People (LEAP) Project, Sexton has analysed the political framing of CM in relation to similar narratives found among emerging plant-based meat companies. Her work documents the narratives through which CM is presented as an edible, and transformative, technology, and how political notions of what constitutes ‘good’ food and ‘good consumers’ are articulated through this ( Sexton, 2016; Sexton, 2018; Sexton *et al.,* 2019).

Over the next couple of years, a number of reports on public perceptions of CM were published. The first was by an international group including staff at the University of Bath ( Marcu *et al.,* 2015). Funded as part of a European Union FP7 project, this work looked at perceptions in the UK, Portugal and Belgium, and found participants’ accounts often connected CM to existing reference points to make sense of it, be that familiar metaphors, science fiction, or their existing opinions on the politics of food technology and commercialisation. A second study, funded by and conducted with the Tyndall Centre for Climate Change Research at the University of Manchester ( O'Keefe *et al.,* 2016), looked at focus group responses to a range of food practices intended to address climate change, including CM. It found consumers did not strongly link food choices to climate change, but were more likely to embrace changes that fit more closely to their existing competencies and practices. More recently, Christopher Bryant, working with the Bath group and funded by the UK Economic and Social Research Funding Council, has published a further set of survey-based consumer analyses ( Bryant & Barnett, 2018). His findings include the observation that consumer positivity towards CM increases when it is called ‘clean meat’ as opposed to ‘lab grown meat’ ( Bryant & Barnett, 2019), and, in work funded by the Animal Advocacy Research Fund, that India and China have higher levels of acceptance for cultured meat than the USA, and higher familiarity predicted higher acceptance ( Bryant *et al.,* 2019). Finally on public perceptions, Professor Frank Vriesekoop of Harper Adams University has worked with a team of international colleagues on consumers perspectives in the UK, Spain, Brazil and the Dominican Republic ( Gómez-Luciano *et al.,* 2019). They found readiness to consume alternative proteins was greater in higher income countries, and that consumers found plant-based proteins more attractive than CM.

Recently, Dr Josh Milburn, of the University of Sheffield, has also written supportively on the ethics of cultured meat and milk. On CM, Milburn argues appropriate safeguards can be put in place to prevent harm to animals from which cells are extracted, and hierarchies between humans and animals can be overcome by using humans as the cell source for CM ( Milburn, 2016). Milburn then argues cultured milk has key differences ethically to CM in that milk is unambiguously food, and never part of an animal’s body. Cultured milk, Milburn argues, should be supported while ensuring it does not stand to legitimate the current milk industry, while again noting that cultured human milk would undermine any concerns about reasserting human-animal hierarchies ( Milburn, 2018).

Finally, John Miller, also of the University of Sheffield and another attendee of the 2014 Rothbury event, is writing on the literary history of CM. He identifies science fiction narratives concerning CM as early as 1881, and analyses in detail a depiction from the 1952 novel ‘The Space Merchants’ ( Pohl & Kornbluth, 1984) that on one hand gestures towards a politics that values traditional meat production over CM, but on the other engages with the problems of capitalism that frame meats produced in any form ( Miller, 2019).

### Environmental life cycle analyses

Life cycle analyses (LCA) assess the environmental impact across the lifecycle of a particular product or output. Such work has inherent difficulties in the context of cellular agriculture as the products being modelled are still early in their research and development process, and have not yet entered scale-up processes. As such LCAs on the topic involve making multiple assumptions, or the use of the closest real-world example in the absence of empirical material on actual cellular agriculture processes. However, potential environmental benefits are key motivators for many cellular agriculture products, so a number of attempts have been made to quantify what this benefit would be. Three of these have been produced in the UK. The earliest of these was the first LCA of CM produced anywhere in the world, by Dr Hannah Tuomisto, then of Oxford University, working with Dr Joost Teixeira De Mattos of the University of Amsterdam. As well as the first, this LCA has to date remained the most optimistic, suggesting that compared to conventionally produced European meat, a CM system could result in 7–45% lower energy use, 78–96% less greenhouse gas emissions, 99% lower land use, and 82–96% lower water use ( Tuomisto & de Mattos, 2011). Four years later, Dr Mark Steer of the University of the West of England conducted an LCA of milk produced through cellular agriculture, based on the work of San Francisco start-up Muufri (now Perfect Day). The modelling here found Muufri’s milk could use 35% of the energy, 16% of the greenhouse gases, 1% of the land and 2% of the water compared to the conventional dairy industry ( Steer, 2015). Finally, more recently in 2019, another Oxford University group published an LCA of CM comparing a wider set of potential production and use contexts than previous work, and looked across multiple timeframes, up until 1,000 years in the future. The study found that, while in many instances CM is climatically superior to conventional livestock production, some scenarios may exist in which this is not the case ( Lynch & Pierrehumbert, 2019).

### 3rd sector groups, charities and think tanks

There are two dedicated UK-based third sector groups in the UK, as well as UK representation of international (generally US-based) groups and a level of interest among UK third sector groups with a broader remit. The first dedicated UK third sector group was ‘Cultivate’, founded in 2016 by an interdisciplinary and cross-sector team of five (that include the authorship team of this review). It describes itself as “a multi-voiced forum intended to support informed dialogue about the emergent field of cellular agriculture from UK perspectives”. It formed after the group who went on to become its founders were invited to a number of discussions at 10 Downing Street about UK policy in this area, during which they produced a review of UK activity and a set of policy recommendations, one of which was to establish the networking group that became Cultivate. A significantly reedited version of their report went on to be published as Stephens *et al.*, (2018). Cultivate have hosted an annual meeting since 2016 and produce written outputs on the topic. The organisation operates without external funding and functions with low costs. Events are supported by attendee entrance fees and founder contributions.

A second dedicated UK third sector group was established in 2018. ‘Cellular Agriculture UK’ seeks to “provide a clear, central hub and contact point for those who have independently developed interest in the space” and to “reach out to potential interested parties and support their engagement in the space” ( Cellular Agriculture, 2018) and held their first activities in early 2019. Beyond these UK-based groups, three US based groups also have UK representation. The Cellular Agriculture Society have UK-based volunteers. The Good Food Institute now has a representative in the UK, having recently employed its first UK-based staff member, Richard Parr, as their Managing Director (EU); as noted previously, they have funded Hanga’s research at Aston University. Another leading US-based third sector group—New Harvest—fund PhD research at the Ellis lab at the University of Bath and Gouveia’s work at Newcastle University, as well as earlier work at King’s College London and the University of Oxford.

Additionally, a number of UK groups with broader focus have produced reports about CM, including the Adam Smith Institute ( Hollywood & Pirie, 2018), the Food Ethics Council (2015) and the Nuffield Council on Bioethics (2019). Among the most detailed is a report by Chatham House ( Froggatt & Wellesley, 2019) that specifically articulates considerations for the EU. In particular, they raise issues relating to how regulation and labelling decisions made by policy-makers could frame future direction and pace of growth.

## Conclusion

Cellular agriculture, both as a term and as a field of activity, remains relatively new. Those developing the technologies associate it with a range of significant benefits, but the technology remains early-stage in many cases, and the capacity of these technologies to deliver these benefits remains unknown and subject to the social context of their introduction. The technologies have garnered support over the last five years from a set of technology investors, often with links to Silicon Valley finances or modes of working. UK activity, in this context, is increasing but remains smaller than that found in countries such as the US, Israel and the Netherlands.

The longest-standing UK work has been university-based. The University of Bath is a leading institution among these, in part as it was an early-mover, and due to its existing track-record in bioprocessing, but also partly due to chance in that Bath has three separate groups (one of which is based in the Department of Psychology, two based in the Department of Chemical Engineering) that have independently chosen to work in this area. More recently the number of companies has been growing. Unlike many of the American, Israeli and Dutch companies, all but one UK company have adopted a business-to-business model, seeking to supply components necessary for CM production to other CM companies. The exception is Higher Steaks, who have adopted the full-stack model of seeking to produce marketable CM products. We also note that all of the UK companies are focused upon CM, with none of them addressing the broader set of cellular agriculture products. These companies are either seeking, or have gained, initial seed funding, but have attracted less finance than the leading companies globally. Also notable is that the UK-based investors have most frequently directed their finance outside of the UK, primarily to the US, and have invested less domestically.

We have demonstrated that the UK has a long history of lively work on the social and policy aspects of cellular agriculture, covering key but diverse topics including the production of meaning, consumer responses, economics and regulatory analysis. This is not, we suggest, anything specific to cellular agriculture, but instead represents a strong UK body of work on the analysis of emerging technologies in general. This academic work has fed into the emerging policy discussion, about how cellular agriculture should be regulated and what role it might have in society if realised as a commercial reality. However, beyond the organisation Cultivate, there are few formal links between the social scientists and those conducting laboratory work

We detailed the two examples of UK cellular agriculture laboratory work beyond CM; the leather work and Manchester University and the MP² Project. As we noted, the MP² Project—focused upon a fermentation-based palm oil system—demonstrates that cellular agriculture approaches need not be limited to animal-derived products and suggests that the common-use definition of cellular agriculture could be expanded to include non-animal agricultural products, including oils.

Overall, our review has shown that the UK is not the leading country in the world in cellular agriculture, but it does have an active and diverse community. Given the knowledge base within relevant fields in the UK, there is also significant potential for this body of work to increase in the coming years. We have provided this review to inform interested parties about who is active and what they are doing in the UK as 2020 begins, both for the benefit of audiences keen to engage in 2020, and to record this moment of emergence for the historical record. The future of cellular agriculture is indeterminate, but it seems likely the UK will continue to be involved in the coming years.

## Data availability

No data are associated with this article.
