# Supplementary material for: Cellular agriculture in the UK: a review
Source: Wellcome Open Res. 2020 Oct 12;5:12. Originally published 2020 Jan 24. [Version 2] doi: 10.12688/wellcomeopenres.15685.2 (PMC7014924; doi:10.12688/wellcomeopenres.15685.2)
Supplement: Peer review history

# Peer review: Reviewer response for version 2

**DOI:** 10.21956/wellcomeopenres.17472.r40838

**Published:** 2020-10-19

**Authors:** Sissel Beate Rønning

**Affiliations:** 1 Nofima AS, Ås, Norway

I have no further comments to make, thank you for this revised version.

Is the review written in accessible language?

Yes

Are all factual statements correct and adequately supported by citations?

Yes

Are the conclusions drawn appropriate in the context of the current research literature?

Yes

Is the topic of the review discussed comprehensively in the context of the current literature?

Yes

Reviewer Expertise:

Food Sciences, cultured meat, muscle cell biology.

I confirm that I have read this submission and believe that I have an appropriate level of expertise to confirm that it is of an acceptable scientific standard.

# Peer review: Reviewer response for version 2

**DOI:** 10.21956/wellcomeopenres.17472.r40839

**Published:** 2020-10-19

**Authors:** Elisabeth Abergel

**Affiliations:** 1 Department of Sociology and Institute for Environmental Science, University of Quebec at Montreal (UQAM), Montreal, Canada

The revised article has addressed a lot of the comments and seems more focused in some parts, most notably the section dealing with social science and the involvement of the University of Bath in Cellular Meat. The authors have included more details and have provided satisfactory responses to reviewers. I, therefore, think the article is ready for indexing.

Is the review written in accessible language?

Yes

Are all factual statements correct and adequately supported by citations?

Yes

Are the conclusions drawn appropriate in the context of the current research literature?

Yes

Is the topic of the review discussed comprehensively in the context of the current literature?

Yes

Reviewer Expertise:

Sociology of science and technology, biotechnology and the bioeconomy, agricultural technologies, sustainable food systems, food future, gender and food, and food ethics.

I confirm that I have read this submission and believe that I have an appropriate level of expertise to confirm that it is of an acceptable scientific standard.

# Peer review: Reviewer response for version 1

**DOI:** 10.21956/wellcomeopenres.17190.r37679

**Published:** 2020-02-14

**Authors:** Mariana Petronela Hanga

**Affiliations:** 1 School of Life and Health Sciences, Aston University, Birmingham, UK

This manuscript is a comprehensive review detailing the cellular agriculture landscape with a focus on the UK. It covers information about current relevant academic research including social science and life cycle analysis, companies acting in this area, private investors and other organisations with an interest. The authors have carried out a thorough investigation covering all stakeholders in the UK, but within the worldwide context. I liked very much that the authors have included a section on global context as it is important to acknowledge that the current leaders in the cellular agriculture scene are USA, Netherlands and Israel, while the UK at the moment is still in the early stages with only one start-up company aiming to commercialize a cultivated meat product and a handful of academic groups actively involved in this area.

 The manuscript starts by defining cellular agriculture as covering 2 approaches: “tissue-engineered based” and “fermentation-based cellular agriculture” providing examples of types of products for each. I liked that a classification of the different approaches to cellular agriculture products was attempted as cultivated meat is often presented in the media, thus ignoring the other types of cellular agriculture products. However, in my opinion, the term “tissue engineered based” is perhaps slightly misleading as it implies only one type of cultivated meat product which is structured meat. It does not cover the non-structured meat ( *e.g*. minced meat) that is obtained without the need of a scaffold specific to tissue engineering approaches. Perhaps a different, more inclusive term would be more appropriate.

 The authors state that the aim of cellular agriculture is “to reduce (or entirely replace) animal agriculture”, however I don’t agree in totality with this statement as it is highly unlikely that complete replacement of animal agriculture will be achieved with the existing capacity. A partial replacement is more realistic. 

 An interesting proposal was made by the authors to extend the current definition of cellular agriculture to include cell-based agricultural goods that are not sourced from animals with a particular reference to fermentation-based palm oil from yeast. This is an interesting concept that falls within a different product category with potential benefits, however I would have liked to read more about these benefits, as there is only a brief statement saying that “this work ties to typical environmental and animal welfare concerns”, but doesn’t explicitly describe those benefits, particularly from the animal welfare point of view. 

 The manuscript is very well written and structured in a logical manner, being very easy to follow from one section to another. The conclusions are meaningful and summarize nicely the findings of this investigation. There is a lack of peer-reviewed published manuscripts in this domain which was acknowledged by the authors. Additionally, the majority of active players in this domain worldwide belong to the private sector and don’t publicize their proprietary technology or the stage of development that they are at. As such, it is difficult to assess the timeline to a marketable cellular agriculture product, it being cultivated meat or other ( *e.g.* leather, milk).

Is the review written in accessible language?

Yes

Are all factual statements correct and adequately supported by citations?

Partly

Are the conclusions drawn appropriate in the context of the current research literature?

Yes

Is the topic of the review discussed comprehensively in the context of the current literature?

Yes

Reviewer Expertise:

Bioprocessing of stem cells, cultivated meat, bioreactors, scale up, cell therapy manufacturing.

I confirm that I have read this submission and believe that I have an appropriate level of expertise to confirm that it is of an acceptable scientific standard.

# Response

**Published:** 2020-05-01

**Authors:** Neil Stephens

**Affiliations:** Brunel University London, UK

We thank the reviewer for these useful and positive comments. We have made three specific edits to the paper in response to the comments.

First, we have clarified our use of “tissue engineering based” cellular agriculture to show that we include non-structured cultured meat within this. We did not want to use a different term as that phrase has already been used in published work elsewhere.

Second, we made a slight edit to acknowledge that partiality of animal agriculture replacement by saying one aim of cellular agriculture is “perhaps entirely replacing” animal agriculture.

Thirdly, we have added a short line on the claimed benefits of a cellular agriculture approach to palm oil production, noting it is: “specifically around deforestation and its impact upon greenhouse gases and biodiversity”.

# Peer review: Reviewer response for version 1

**DOI:** 10.21956/wellcomeopenres.17190.r37682

**Published:** 2020-02-11

**Authors:** Mark J. Post, Joshua Flack

**Affiliations:** 1 Mosa Meat, B.V., Maastricht, The Netherlands; 2 Department of Physiology, Maastricht University, Maastricht, The Netherlands

This review provides a timely record of the current state of the Cellular Agriculture field in the United Kingdom. As the authors surmise, the UK is a leading nation in terms of scientific research, but arguably has been somewhat ‘behind the curve’ in terms of Cellular Agriculture. As the authors detail, this is now changing, although the developments in the UK are more below the radar than the activities in the US, Israel and several European nations, such as the Netherlands.

 The overview constitutes a useful resource for those looking for an entry point into understanding the UK-based Cellular Agriculture landscape. It provides a good cross-sectional view of all activities in this space in the UK, with some comparison to other nations.

 The authors struggle with the inclusivity of the term cellular agriculture, more specifically whether to include all biotechnology using recombinant technology in micro-organisms, i.e. fermentation. The existing industry in this space, that historically predates cellular agricultural activities by some decades and which focusses on many applications in addition to agricultural products and food, will likely not recognize itself in this qualification. Fortunately, to date there is no practical confusion over the term cellular agriculture, but with advancing technologies and the emergence of cross-over technologies as well as applications, the term will become less defining.

 As in most other countries, the rapid emergence of cellular agriculture in the UK, depends on privately funded initiatives in the absence of public funding. As reported, there were some government initiatives to get a clear understanding of the field during the Cameron administration, but this apparently has faded in the wake of government changes and obvious focus on other subjects. In various countries on the continent and indeed in Brussels itself, consultations have started and political views are being formed, hopefully followed by funding or permissive legislative activities. In this respect it will be interesting and challenging how the recent political developments in the UK will affect the current or future collaborative projects in the Cellular Agriculture space with European partners. This will especially be important for future regulation. Europe has been leading the way in defining how cellular agriculture products, specifically cultured meat, will be regulated. It is unknown how the UK will manage regulation of novel food products in the near future. The authors might want to speculate or have insight into what is a likely regulatory scenario.

 Nevertheless, it is encouraging to learn that there are many academic groups involved in cellular agriculture, one way or the other. This is attested by the relatively large number of scientific publications coming out of UK academic groups, a majority of them on social science aspects. On the laboratory science level, it should be mentioned that the Dutch start-up Meatable was co-founded by a UK-based academic, Mark Kotter, and is making use of stem cell technologies developed in his laboratory in Cambridge.

 The discussion related to the ‘full-stack’ nature of Higher Steaks as a Cellular Agriculture company is an interesting point, and one that might warrant further discussion should the authors wish. Do the authors foresee that such vertically integrated companies will ever come to dominate the UK-based Cellular Agriculture scene? Vertical integration seems to be a business model from the past and the current attempts to vertical integration by startup companies should be viewed as the result of a lack of suppliers active in this space. It would therefore be good to define what the strength of UK-based companies or academic groups is and how they would fit in an eventual cellular agricultural industry. 

 In this rapidly changing field, any publication is inevitably out of date at the time of publication. For example, Memphis Meats recently announced a large Series B funding round ($161M). 

 The authors are in an excellent position to suggest future paths that Cellular Agriculture research in the UK might take, and what strategies should be employed to maximize the efficacy of the current projects and thus improve or accelerate the growth of Cellular Agriculture in the UK and the rest of the world.

Is the review written in accessible language?

Yes

Are all factual statements correct and adequately supported by citations?

Yes

Are the conclusions drawn appropriate in the context of the current research literature?

Yes

Is the topic of the review discussed comprehensively in the context of the current literature?

Yes

Reviewer Expertise:

Tissue engineering; cultured meat; Physiology.

We confirm that we have read this submission and believe that we have an appropriate level of expertise to confirm that it is of an acceptable scientific standard.

# Response

**Published:** 2020-05-01

**Authors:** Neil Stephens

**Affiliations:** Brunel University London, UK

We thank the reviewers for their comments on the paper. We acknowledge that the paper was published just prior to Memphis Meat’s $161 Series B, but we have not included this as the review is intended to be accurate as of the end of 2019.

We have edited the paper following this review in that we have now included a paragraph about Mark Kotter’s work with Meatable, and we thank the reviewers for highlighting this to us.

# Peer review: Reviewer response for version 1

**DOI:** 10.21956/wellcomeopenres.17190.r37683

**Published:** 2020-02-06

**Authors:** Elisabeth Abergel

**Affiliations:** 1 Department of Sociology and Institute for Environmental Science, University of Quebec at Montreal (UQAM), Montreal, Canada

The article sets out to provide an inventory of the field of Cellular Agriculture in the UK including the private and public researchers and companies, investors as well as a very broad overview of the types of products they are developing. There is also a section on social science, which aims to situate how academics studied and were instrumental in opening up various aspects of CM in several disciplinary contexts such as sociology, bioethics, law, political framing, environmental assessments, etc.

 There is a very synthetic but well crafted introduction which retells the story of CM and includes some notable UK references, notably Winston Churchill's famous quotation as well as the highly publicized tasting of the first "lab-grown burger" which took place in London.

 The paper highlights current and recent developments in the UK within the Cell Agriculture space in relations to other countries (US, Holland and Israel for instance). The authors also describe three studies of the environmental impact assessments of potential Cellular Agriculture systems emanating from the UK. Hence, readers get a snapshot of the UK's involvement in and contributions to Cellular Agriculture since its beginning until the end of 2019. 

 The article is very descriptive and offers very little analytical perspective as is expected from the authors' stated objectives. In terms of the review methodology and the purpose of the article, the contents are coherent and follow a logical order. The authors are extremely knowledgeable in the field of Cellular Agriculture in the UK on the scientific and social science side. They have been involved in this issue since its beginnings in the UK and internationally. Their involvement in the field is acknowledged in the review methodology.

 However, one cannot help question the purpose of this paper from an academic and scholarly point of view. While it spans, it seems, a great variety of actors and players in Cellular Agriculture, it lacks in depth and possibly critical distance as the authors feature prominently in the article. It is on some level very self-referential. One also wonders who the intended target audience might be as the article provides some technical details of scientific developments but leaves out broader information about key findings of studies from its "portfolio" of social science research. 

 For instance: 

- While the paper discusses scientific research and social sciences contribution to the field, what are the links between these communities, if any (besides the Cultivate organization and the authors)?
- One would have liked to have known the role of UK scientists, entrepreneurs and/or investors who promote Cellular Agriculture at the international level through association meetings, start-up events and conferences? The scope of influence of people like Illtud Dunsford for example (Cellular Agriculture Ltd) who represents the farmer's perspective at CM events organized outside the UK (he is mentioned in the paper as coming from a farming background but he is also partnered with M.E. one of the authors).
- What are the ties between university researchers and private companies in terms of shared levels of expertise, within the UK and outside the UK and in terms of funding besides funding doctoral students?
- Does the UK demarcate itself in terms of expertise, technologies, areas of research, patented technologies, expected products, infrastructure, etc.? Why is the University of Bath such a leader? 
- What might be occurring in terms of government involvement? What regulatory developments, if any?
- How and who funded the social sciences research conducted in the UK? Who funds Cultivate (created by both authors as a forum for discussing UK cellular agriculture)?
- Who are the institutional actors and related industries supporting cellular agriculture in the UK? And what roles do they play in the development of the field?

The paper reads almost as a promotional piece, it doesn't delve into deeper questions. It is, as stated earlier, an inventory of sorts. One of the interesting points raised in the article is the brief discussion about potentially expanding the definition of cellular agriculture to include non-animal sourced products. This could have been discussed further as definitions and language are very strategic in Cellular Agriculture but also, to not leave readers with the impression that including fermentation based palm oil production was placed there because it is an active area of research in the UK. It raises questions about how far the definition of Cellular Agriculture can be expanded.

Is the review written in accessible language?

Yes

Are all factual statements correct and adequately supported by citations?

Yes

Are the conclusions drawn appropriate in the context of the current research literature?

Yes

Is the topic of the review discussed comprehensively in the context of the current literature?

Yes

Reviewer Expertise:

Sociology of science and technology, biotechnology and the bioeconomy, agricultural technologies, sustainable food systems, food future, gender and food, and food ethics.

I confirm that I have read this submission and believe that I have an appropriate level of expertise to confirm that it is of an acceptable scientific standard, however I have significant reservations, as outlined above.

# Response

**Published:** 2020-05-01

**Authors:** Neil Stephens

**Affiliations:** Brunel University London, UK

We thank the reviewer for their thoughtful comments, and we have made a number of improvements to the paper in response. We respond to each of the major points in turn below.

- one cannot help question the purpose of this paper from an academic and scholarly point of view. While it spans, it seems, a great variety of actors and players in Cellular Agriculture, it lacks in depth and possibly critical distance as the authors feature prominently in the article. It is on some level very self-referential. One also wonders who the intended target audience might be as the article provides some technical details of scientific developments but leaves out broader information about key findings of studies from its "portfolio" of social science research.

This critique mirrors the comments of another of the reviewers, and we have responded by extending the opening paragraph to give a clear statement of what the paper aims to achieve, and who we think will benefit from it. In terms of purpose, for us, the paper is one stage in a set of activities that we are engaged in that will support a deeper and more critically engaged discussion about cellular agriculture in the UK. In this regard this paper is very important to us as a baseline for a broader discussion. Finally on this, one reason this journal is a good fit for this specific paper is it requirements in terms of purpose: “All new findings supported by original source data are welcome, regardless of the perceived interest and the extent of novelty”.

- While the paper discusses scientific research and social sciences contribution to the field, what are the links between these communities, if any (besides the Cultivate organization and the authors)?

We are not aware of any further links, but we cannot definitively say there are none. We have added a line to the paragraph in the conclusion about the social science work on CM noting that these links are limited.

- One would have liked to have known the role of UK scientists, entrepreneurs and/or investors who promote Cellular Agriculture at the international level through association meetings, start-up events and conferences? The scope of influence of people like Illtud Dunsford for example (Cellular Agriculture Ltd) who represents the farmer's perspective at CM events organized outside the UK (he is mentioned in the paper as coming from a farming background but he is also partnered with M.E. one of the authors).

This paper was not intended to detail the activities of UK groups in the international context, or judge their influence (although we do note the role of UK investors in non-UK companies). So we have not addressed this point as it is beyond the paper’s remit.

- What are the ties between university researchers and private companies in terms of shared levels of expertise, within the UK and outside the UK and in terms of funding besides funding doctoral students?

The paper identifies multiple ties between universities and private companies. Cellular Agriculture Ltd is developing technology from the University of Bath, CellulaREvolution is developing technology from Newcastle University, Higher Steaks is working with the University of Edinburgh, and (featured in this revised version) Meatables is commercialising technology from Cambridge University.

- Does the UK demarcate itself in terms of expertise, technologies, areas of research, patented technologies, expected products, infrastructure, etc.? Why is the University of Bath such a leader?

This is an interesting question but we do not believe that ‘the UK’ is close enough to a singular organised group that does actively demark or self-define itself. As the paper shows, activity in the UK is characterised by a relatively small number of groups, many of which are relatively early in their operations. We have added a new line to the conclusion that comments on the University of Bath: “The University of Bath is a leading institution among these, in part as it was an early-mover, and due to its existing track-record in bioprocessing, but also partly due to chance in that Bath has three separate groups (one of which is based in the Department of Psychology, two based in the Department of Chemical Engineering ) that have independently chosen to work in this area”.

- What might be occurring in terms of government involvement? What regulatory developments, if any?

The government has said very little formally on this topic. As noted in the new introduction, this paper has been produced in part to allow stakeholders such as the government to more easily identify activity in the UK in anticipation of possible future policy discussion in the UK.

- How and who funded the social sciences research conducted in the UK?

Following this comment and that of another reviewer, the section on UK social science has been edited substantially to both increase the coverage of the content of the social science work, and identify its funding sources where they are known. However, this has been done while working to retain the succinct overview quality of the paper.

- Who funds Cultivate (created by both authors as a forum for discussing UK cellular agriculture)?

The paragraph overviewing Cultivate now includes these details: “The organisation operates without external funding and functions with low costs. Events are supported by attendee entrance fees and founder contributions.”

- Who are the institutional actors and related industries supporting cellular agriculture in the UK? And what roles do they play in the development of the field?

We are a little confused by this comment, as the paper primarily functions to list the key institutional actors and the role they play in the development of the field, so we feel this is already addressed across the paper.

Thanks again for your comments, we hope you agree this is now an improved manuscript.

# Peer review: Reviewer response for version 1

**DOI:** 10.21956/wellcomeopenres.17190.r37681

**Published:** 2020-02-03

**Authors:** Sissel Beate Rønning

**Affiliations:** 1 Nofima AS, Ås, Norway

This manuscript gives an overview of the cellular agriculture in UK, and it presents information on a relatively unexplored subject where publication of data is still limited. The manuscript summarizes nicely the research activity, and the commercial activities in the UK, and I hope the authors will extend this work to include rest of Europe in their next review. As the authors are among the most active researchers in the UK on the topic, this unfortunately implies they review and refer to much of their own work. This is a limitation of the manuscript, and although the authors address this in the review methodology the authors must be careful to provide a balanced and comprehensive overview of the research field. I think this manuscript is important, well performed and I recommend it for indexing if the authors address my comments.

 Comments:

- Could the authors emphasize even more the objective of this review? Why is it necessary with this review and most importantly: who is it for? 
- I am surprised there is such limited research (and investments) on fermentation-based cell agriculture in UK. According to reports such as the RethinkX report, these types of products are more likely to reach the market sooner compared with tissue-based cell agriculture. Perhaps the authors could comment on this interesting fact? Also, in this report the authors predict a total collapse of food production as we see it today, and how to handle post-animal production is not something we should ignore in the future. Who will/should produce cultured meat (local farmers, agribusiness, bioscientists, pharmaceutical business)? What effect will synthetic protein production/cellular agriculture have on the conventional agricultural sector? How to prepare for its arrival? And what about other markets, such as production of growth factors, fish feed, pet food? According to White Paper from World Economic Forum in 2019, “public support for alternative proteins will most likely be suppressed if the social costs of their adapter are seen to high”. Perhaps some of these questions could be addressed in this manuscript?
- The text is sometimes incoherent and unfocused. For example, a short historical introduction to cell agriculture is given in the beginning of introduction. Then another historical section, describing Churchill and the first burger is described in another chapter; cell agriculture-related work in UK. In my opinion this could be moved to the introduction, keeping the chapters more focused.
- I missed information on the technology transfer between medical field and the field of cell agriculture. i.e. production of vaccines, tissue engineering, production of insulin and so on. What is similar, and what differs?
- The authors point out that “substantial technical hurdles remain”. In order to help the reader, could the authors perhaps describe briefly those challenges, not just refer to them? 
- In my opinion the readability of the manuscript will increase with tables and graphics.
- Obviously, Stephens is an expert in social science, and I think the manuscript will benefit from extending the chapter on social science to not just mention all the studies and reports of social sciences, but also the results and conclusions of these works.

Is the review written in accessible language?

Yes

Are all factual statements correct and adequately supported by citations?

Yes

Are the conclusions drawn appropriate in the context of the current research literature?

Yes

Is the topic of the review discussed comprehensively in the context of the current literature?

Yes

Reviewer Expertise:

Food Sciences, cultured meat, muscle cell biology.

I confirm that I have read this submission and believe that I have an appropriate level of expertise to confirm that it is of an acceptable scientific standard, however I have significant reservations, as outlined above.

# Response

**Published:** 2020-05-01

**Authors:** Neil Stephens

**Affiliations:** Brunel University London, UK

Thank you for your review of our paper. We have edited the text to address the issues raised. Below we detail our response to the core comments in turn:

- Could the authors emphasize even more the objective of this review? Why is it necessary with this review and most importantly: who is it for?

We have more than doubled the length of the opening paragraph to state explicitly the purpose of the review. We also note that we believe it will be useful for diverse actors within the UK policy and practice as a building block for subsequent discussion. We plan to use the review as a resource in future work we will be conducting in this context ourselves.

- I am surprised there is such limited research (and investments) on fermentation-based cell agriculture in UK. According to reports such as the RethinkX report, these types of products are more likely to reach the market sooner compared with tissue-based cell agriculture. Perhaps the authors could comment on this interesting fact? Also, in this report the authors predict a total collapse of food production as we see it today, and how to handle post-animal production is not something we should ignore in the future. Who will/should produce cultured meat (local farmers, agribusiness, bioscientists, pharmaceutical business)? What effect will synthetic protein production/cellular agriculture have on the conventional agricultural sector? How to prepare for its arrival? And what about other markets, such as production of growth factors, fish feed, pet food? According to White Paper from World Economic Forum in 2019, “public support for alternative proteins will most likely be suppressed if the social costs of their adapter are seen to high”. Perhaps some of these questions could be addressed in this manuscript?

We do not have a robust answer to the question of why there is limited fermentation-based cellular agriculture in the UK. The core role of this paper is simply to note that this is the case. It is also beyond the remit of this paper to devise an account of what impact cellular agriculture may have on food production and other markets going forward. However, as we noted above and in the edited introduction, in the UK context we hope this paper will be one step in supporting an informed discussion that can start to address these issues.

- The text is sometimes incoherent and unfocused. For example, a short historical introduction to cell agriculture is given in the beginning of introduction. Then another historical section, describing Churchill and the first burger is described in another chapter; cell agriculture-related work in UK. In my opinion this could be moved to the introduction, keeping the chapters more focused.

We have moved this text as suggested, and agree it improves the paper.

- I missed information on the technology transfer between medical field and the field of cell agriculture. i.e. production of vaccines, tissue engineering, production of insulin and so on. What is similar, and what differs?

This is an interesting topic, but we feel this is beyond the focus of this particular paper in terms of the objectives it has set out.

- The authors point out that “substantial technical hurdles remain”. In order to help the reader, could the authors perhaps describe briefly those challenges, not just refer to them?

We have added a brief description as requested, in which we note: “This includes producing culture media at a lower cost;, developing cell lines that have the capacity for indefinite propagation and have a high culture-efficiency to maximise product yield;, developing reliable automation and bioreactor systems to enable scale-up that is net-zero carbon;, and reproducing the taste, texture and nutritional profile of familiar peat products”.

- In my opinion the readability of the manuscript will increase with tables and graphics.

We have decided not to include any additional tables or graphics, as we were unable to think of what data would appropriately and succinctly be captured in table form.

- Obviously, Stephens is an expert in social science, and I think the manuscript will benefit from extending the chapter on social science to not just mention all the studies and reports of social sciences, but also the results and conclusions of these works.

This section has been extended substantially, more than doubling the text to provide further information on the context of these social science projects and signposting some key findings. It would be possible to expand further on each example, however we believe the current form maintains the right balance between detail and succinctness.
